# Kartogenin inhibits pain behavior, chondrocyte inflammation, and attenuates osteoarthritis progression in mice through induction of IL-10

**DOI:** 10.1038/s41598-018-32206-7

**Published:** 2018-09-14

**Authors:** Ji Ye Kwon, Seung Hoon Lee, Hyun-Sik Na, KyungAh Jung, JeongWon Choi, Keun Hyung Cho, Chang-Yong Lee, Seok Jung Kim, Sung-Hwan Park, Dong-Yun Shin, Mi-La Cho

**Affiliations:** 1 The Rheumatism Research Center, Catholic Research Institute of Medical Science, College of Medicine, The Catholic University of Korea, Seoul, Republic of Korea; 2 Impact Biotech, Korea 505 Banpo-Dong, Seocho-Ku, 137-040 Seoul, Korea; 3 College of Pharmacy, Gachon University, 191 Hambakmoe-ro, Yeonsu-gu, Incheon, 406-799 Korea; 4 Department of Orthopedic Surgery, Uijeongbu St. Mary’s Hospital, College of Medicine, The Catholic University of Korea, Seoul, Korea; 5 Division of Rheumatology, Department of Internal Medicine, Seoul St. Mary’s Hospital, College of Medicine, The Catholic University of Korea, Seoul, South Korea; 6 Department of Biomedicine & Health Sciences, College of Medicine, The Catholic University of Korea, 222, Banpo-daero, Seocho-gu, Seoul, 06591 Republic of Korea

## Abstract

Osteoarthritis (OA) is a major degenerative joint condition that causes articular cartilage destruction. It was recently found that enhancement of chondroclasts and suppression in Treg cell differentiation are involved in the pathogenesis of OA. Kartogenin (KGN) is a small drug-like molecule that induces chondrogenesis in mesenchymal stem cells (MSCs). This study aimed to identify whether KGN can enhance severe pain behavior and improve cartilage repair in OA rat model. Induction of OA model was loaded by IA-injection of MIA. In the OA rat model, treatment an intra-articular injection of KGN. Pain levels were evaluated by analyzing PWL and PWT response in animals. Histological analysis and micro-CT images of femurs were used to analyze cartilage destruction. Gene expression was measured by real-time PCR. Immunohistochemistry was analyzed to detect protein expression. KGN injection significantly decreased pain severity and joint destruction in the MIA-induced OA model. KGN also increased mRNA levels of the anti-inflammatory cytokine IL-10 in OA patients’ chondrocytes stimulated by IL-1β. Decreased chondroclast expression, and increased Treg cell expression. KGN revealed therapeutic activity with the potential to reduce pain and improve cartilage destruction. Thus, KGN could be a therapeutic molecule for OA that inhibits cartilage damage.

## Introduction

Osteoarthritis (OA) is an articular degenerative disease, characterized by chronic pain, joint inflammation, and movement limitations. Since OA is the most common form of arthritis, it causes an excessive economic burden and disrupts quality of life^1,2^. Proinflammatory cytokines and matrix metalloproteinases (MMPs) perform an important role in the pathogenesis of OA. It has been demonstrated that the inflammatory response and MMP activity aggravate OA severity through induction of cartilage degradation^3,4^. Moreover, osteoclastogenesis is associated with OA pathogenesis. There is a large amount of evidence supporting association of osteoclasts with upregulation of bone loss and the increase in tartrate-resistant acid phosphatase (TRAP)-positive cells in the rat joints of OA patients and OA animal models^5,6^.

Lately, the notion of OA pathogenesis has been categorized as an immune inflammatory response. It has been suggested that the inhibition of proinflammatory cytokines could be a therapeutic strategy in experimental OA^7^. It is well reported that the expression of proinflammatory cytokines such as tumor necrosis factor (TNF)-α and interleukin (IL)-6 increases in OA articular cartilage or synovial fluid^8,9^. On the other hand, regulatory T (Treg) cell differentiation and IL-10 levels were reduced significantly in peripheral blood or synovial fluid from OA patients compared to healthy controls or rheumatoid arthritis patients^10^.

Kartogenin (KGN) is a small heterocyclic molecule that enhances chondrocyte differentiation of primary human mesenchymal stem cells (MSCs) through upregulation of chondrogenic gene expression and characteristic chondrocyte activities^11^. Although intra-articular (IA) injection of KGN showed cartilage regeneration in a mouse OA model, KGN has not been studied to improve OA through the inhibition of osteoclastogenesis and upregulation of Treg cell differentiation and IL-10 levels.

We hypothesized that KGN IA injection could ameliorate experimental OA development. To test our hypothesis, we injected KGN intra-articularly into experimental OA *in vivo* models to investigate whether the therapeutic activity of KGN would reduce cartilage destruction and joint inflammation. We also conducted *in vitro* experiments to determine whether KGN could inhibit osteoclastogenesis and induce Treg cell differentiation.

## Results

### KGN controlled pain on the dorsal root ganglion of MIA-induced OA Rats

**Figure 1:**
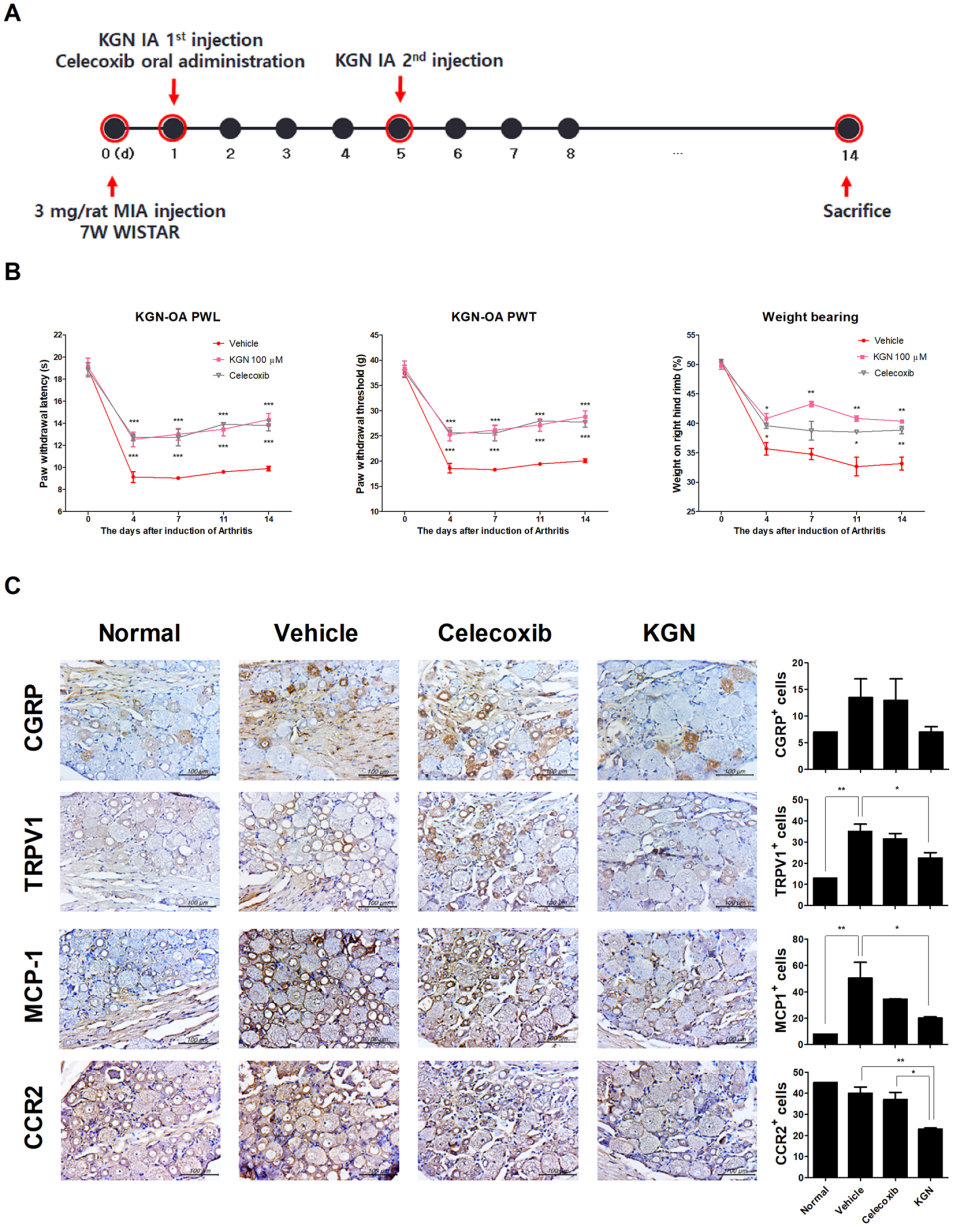
Therapeutic effect of KGN in an initial stage of MIA model. KGN decreased pain and inflammatory factors in the dorsal root ganglion from an OA rat. (**A**) OA induction by IA injection monosodium iodoacetate into Male Wistar rats. Rats were treated with intra-articular injection of KGN (100 μM) or saline (vehicle), 1 and 5 days after MIA injection. Celecoxib (12.5 mg/rat) was given daily by oral administration to MIA-induced rats. (**B**) Conducting experiment mechanical pain were analyzed PWL and PWT on the paw. The right patellar weight-bearing asymmetry in rat models of osteoarthritis is also shown. (**C**) IHC staining was analyzed to measurement the level of CGRP, TRPV1, MCP-1, and CCR2 in the dorsal root ganglion (DRG) of OA rats. Tissue analysis was carried out two times in 3 animal models. These experiments were performed in five individual animal experiments. *P < 0.05, **P < 0.001, ***P < 0.005, comparison to vehicle. Data are representative of three experiments.

To examine whether KGN would reduce pain in OA contrast vehicle (saline) with celecoxib, we performed secondary tactile allodynia in monosodium iodoacetate-induced OA rats. Treatment with KGN and celecoxib raised PWL (paw withdrawal latency and PWT (paw withdrawal threshold) compared to MIA-induced OA group of rats without treatment. Weight bearing was also promoted by treatment with KGN and celecoxib in OA rats (Fig. 1B). To investigate the effect of KGN on the dorsal root ganglion (DRG) of OA rats, we performed immunochemical staining for pain and inflammation markers (Fig. 1C). The level of CGRP decreased in dorsal root ganglion of KGN-treated rats compared to MIA-treated rats (Supplementary Fig. 1). The expression of CGRP, TRPV1, MCP-1, and CCR2 was decreased in the KGN-treated OA rats. These results suggest that KGN and celecoxib treatment improved weight bearing, PWL and PWT in rat with MIA induced OA, while reducing inflammation.

### KGN attenuated cartilage destruction in MIA-induced OA rats

**Figure 2:**
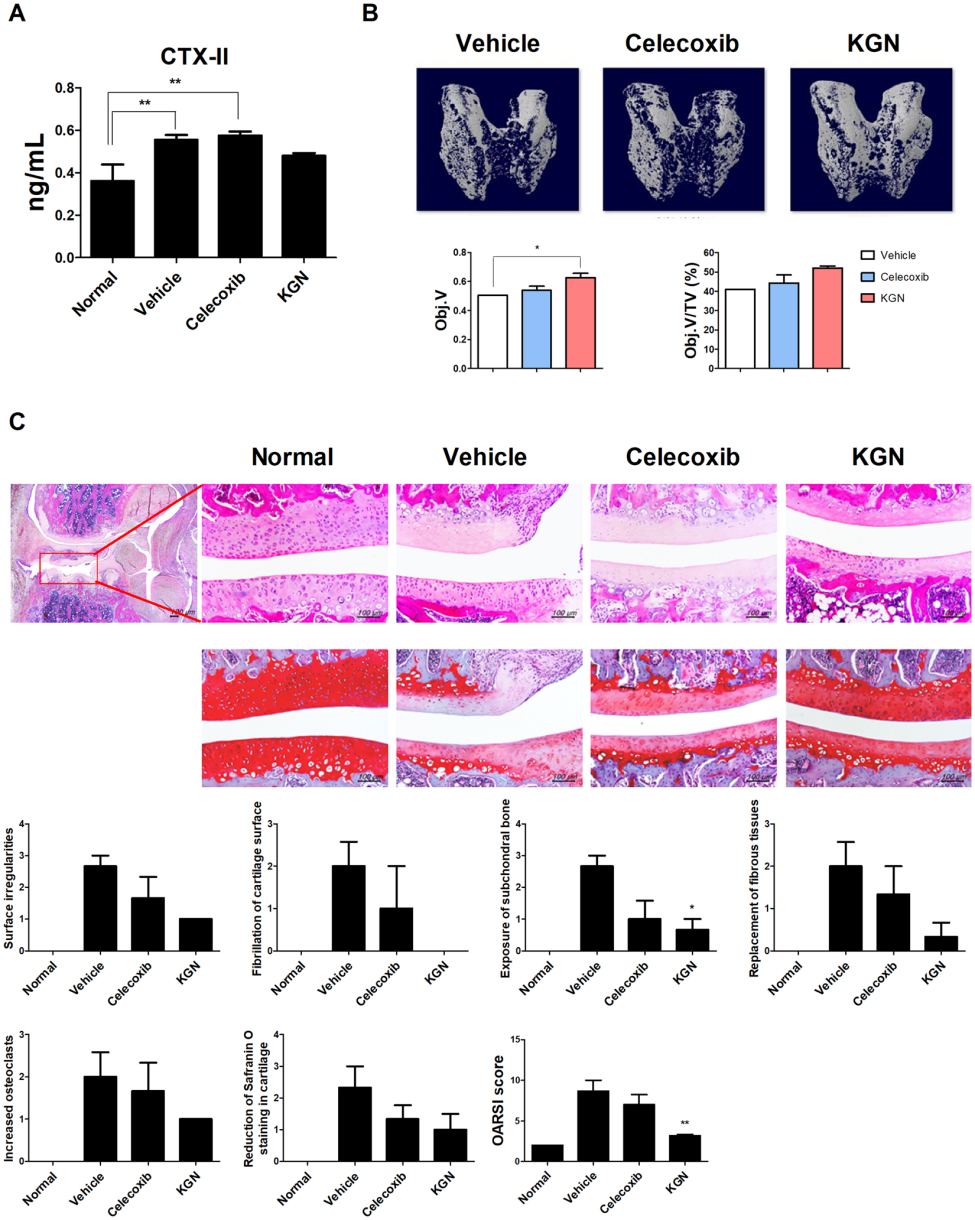
Degenerative disorder of the joint after treatment with KGN in OA model. (**A**) Serum sampling was made at 14 d after MIA injection in rats. Serum CTX-II levels were analyzed by enzyme-linked immunosorbent assay (ELISA). (**B**) Representative micro computed tomography (CT) appearances of the femur condyles at 14 d OA rat models. TV and Obj.V of micro-CT images from femur. (**C**) Histologic examination of the cartilage from rat with OA in each group. Tissue analysis was carried out two times in three animal models. These experiments were performed in 5 individual animal experiments. *P < 0.05, **P < 0.001, comparison to vehicle.

The serum CTX-II (C-telopeptide of type II collagen) level in OA rats was investigated using an ELISA. KGN-treated rats had decreased CTX-II levels compared to vehicle treated rats. However, the CTX-II level in vehicle- and celecoxib-treated rats were significantly higher compared to normal non-OA rats (Fig. 2A).

A representative 3-D reconstruction of subchondral bone from micro-CT images revealed changes in the microstructure of the femur in rat with MIA. KGN showed a protective effect in the femur. Quantitative micro-CT results showed that KGN-treated group improved Obj.V/TV (Objective volume/Total volume) and Obj.V (Objective volume) (Fig. 2B). Conversely, the femurs of MIA-induced OA rats without treatment were degraded and denser. While celecoxib decreased pain severity, the celecoxib-treated group showed degradation similar to that of the vehicle group.

To determine the therapeutic effect of KGN to ameliorate cartilage damage in OA contrast vehicle with celecoxib treatment, we excised the joint and conducted H&E and Safranin O staining. Width and proteoglycan of cartilage component in right knees of osteoarthritis rats treated with KGN and celecoxib decreased in comparison to vehicle. Furthermore, KGN and celecoxib-treated groups all indicated lower cartilage degradation than the vehicle-treated groups as seen from OA histopathological analysis (Fig. 2C).

### KGN increased TIMP-3 and IL-10 expression

**Figure 3:**
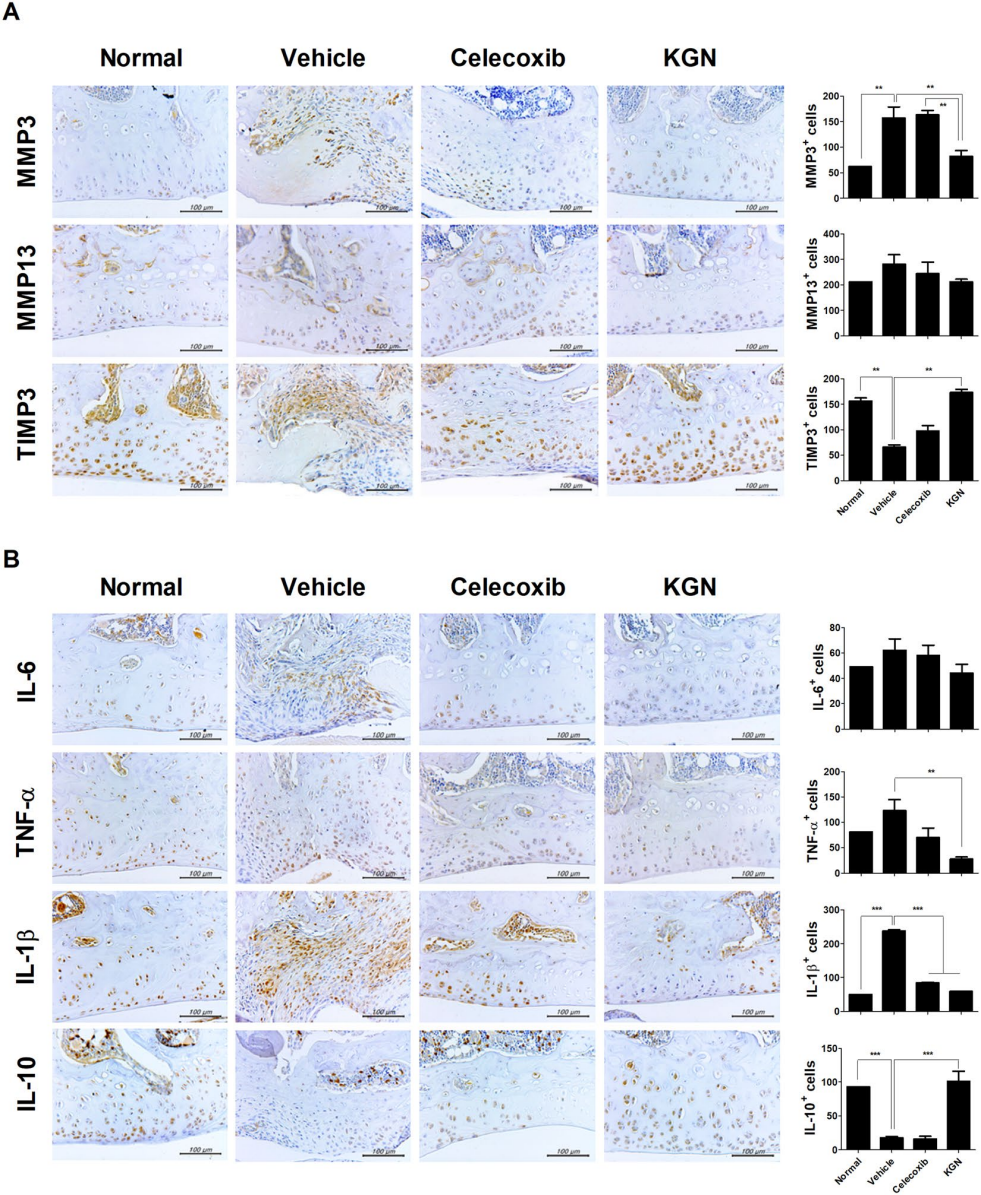
Histopathology of osteoarthritis of a cartilage and chondroclastic (osteoclastic) activity after treatment with KGN in the rat MIA model. (**A**) The level of MMP 3, -13 and TIMP3 were measured in cartilage. TIMP3 expression was increased by KGN. (**B**) The expression of cytokines such as interleukin-6, -1β, TNF-α and IL-10 in cartilage is shown. Decreased expression of IL-6, IL-1β, TNF-α and was induced by KGN and upregulation of IL-10 in cartilage of MIA-injected rats is shown. Tissue analysis was carried out two times in three animal models. These experiments were performed in 5 individual animal experiments. **P < 0.001, ***P < 0.005.

Treatment with KGN produced the most significantly increased expression of TIMP3 and IL-10 compared to vehicle- and celecoxib-treated rats. KGN also decreased the expression of MMP3 and MMP13. On the other hand, TIMP-3 expression was upregulated by treatment with KGN, which was similar to normal levels (Fig. 3A). Furthermore, treatment with KGN and celecoxib reduced the expression of IL-6, TNF-α and IL-1β to below normal levels of expression; however, IL-10 production was enhanced significantly with KGN reaching normal expression levels (Fig. 3B).

### KGN upregulated gene expression of anti-inflammatory cytokines in chondrocytes of human OA patients

**Figure 4:**
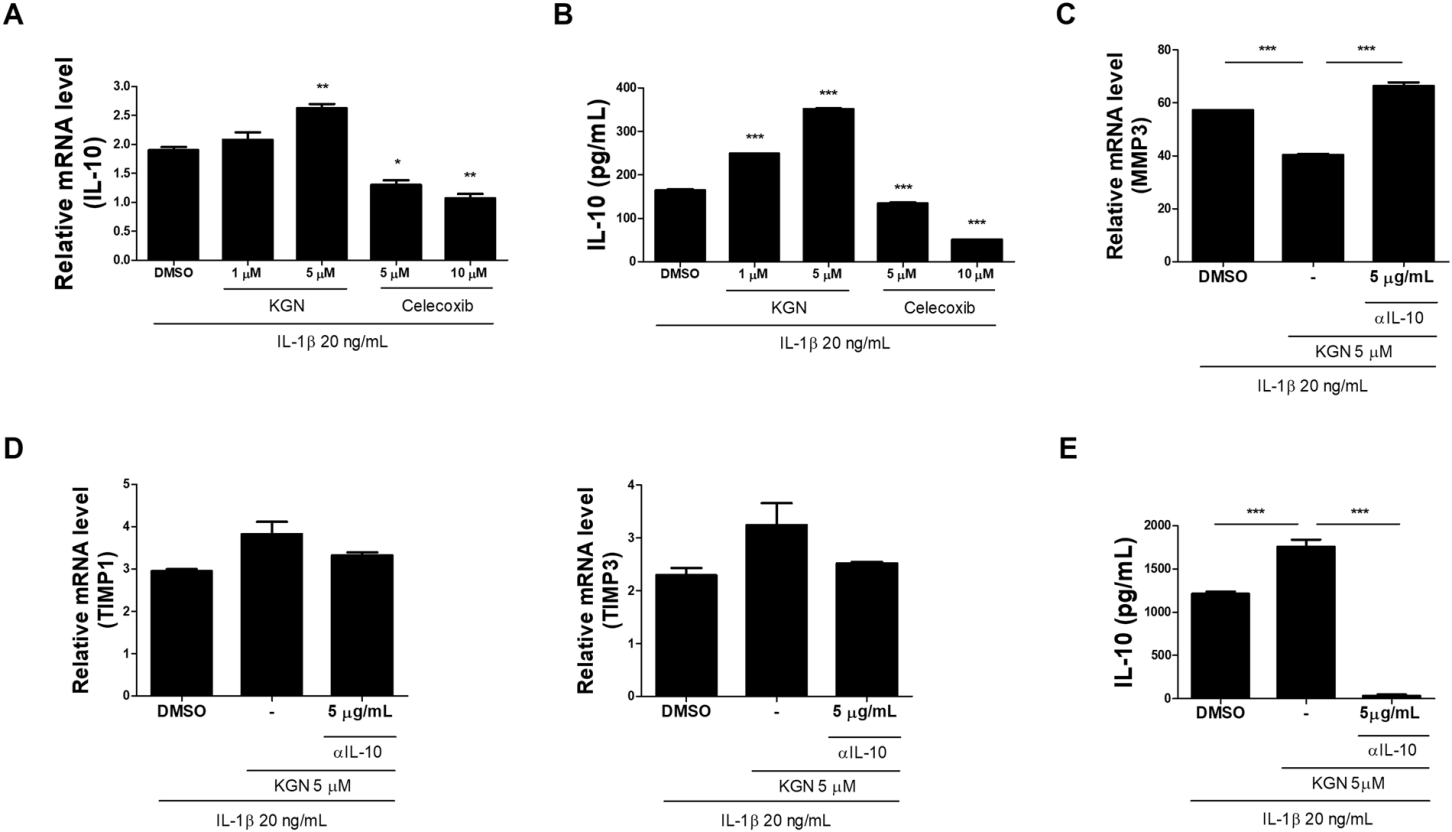
KGN upregulates the expression of anti-inflammatory cytokine IL-10. (**A**) Human OA chondrocytes were incubated with interleukin-1β in the treatment of KGN for 2 days. The level of IL-10 anti-inflammatory cytokine gene was increased in OA chondrocytes after treatment with KGN. (**B**) IL-10 concentrations in culture supernatants were measured by using ELISA. (**C**,**D**) The mRNA levels of catabolic mediators, such as MMP3, and inhibitors, such as TIMP1, 3 were measured in OA chondrocytes after treatment with KGN. (**E**) The expression of IL-10 was measured by using ELISA. *P < 0.05, **P < 0.001, ***P < 0.005, compared to the DMSO-treated group.

To evaluate the ability of KGN to induce anabolic and anti-inflammatory activity in human OA, KGN upregulated levels of anti-inflammatory marker and anabolic factor by real-time polymerase chain reaction. KGN treatment significantly upregulated levels of anti-inflammatory cytokine, such as IL-10, in chondrocytes of human OA patients stimulated with IL-1β (20 ng/mL) (Fig. 4A). Moreover, IL-10 concentrations in culture supernatants were also increased by treatment with KGN in chondrocytes from OA patients compared to celecoxib treatment (Fig. 4B). The relative level of MMP3 mRNA in KGN-treated OA chondrocytes was lower than that of DMSO-treated OA chondrocytes in a IL-10-dependent manner (Fig. 4C) while TIMP 1, 3 increased (Fig. 4D).

### KGN inhibited chondroclast differentiation and activation

**Figure 5:**
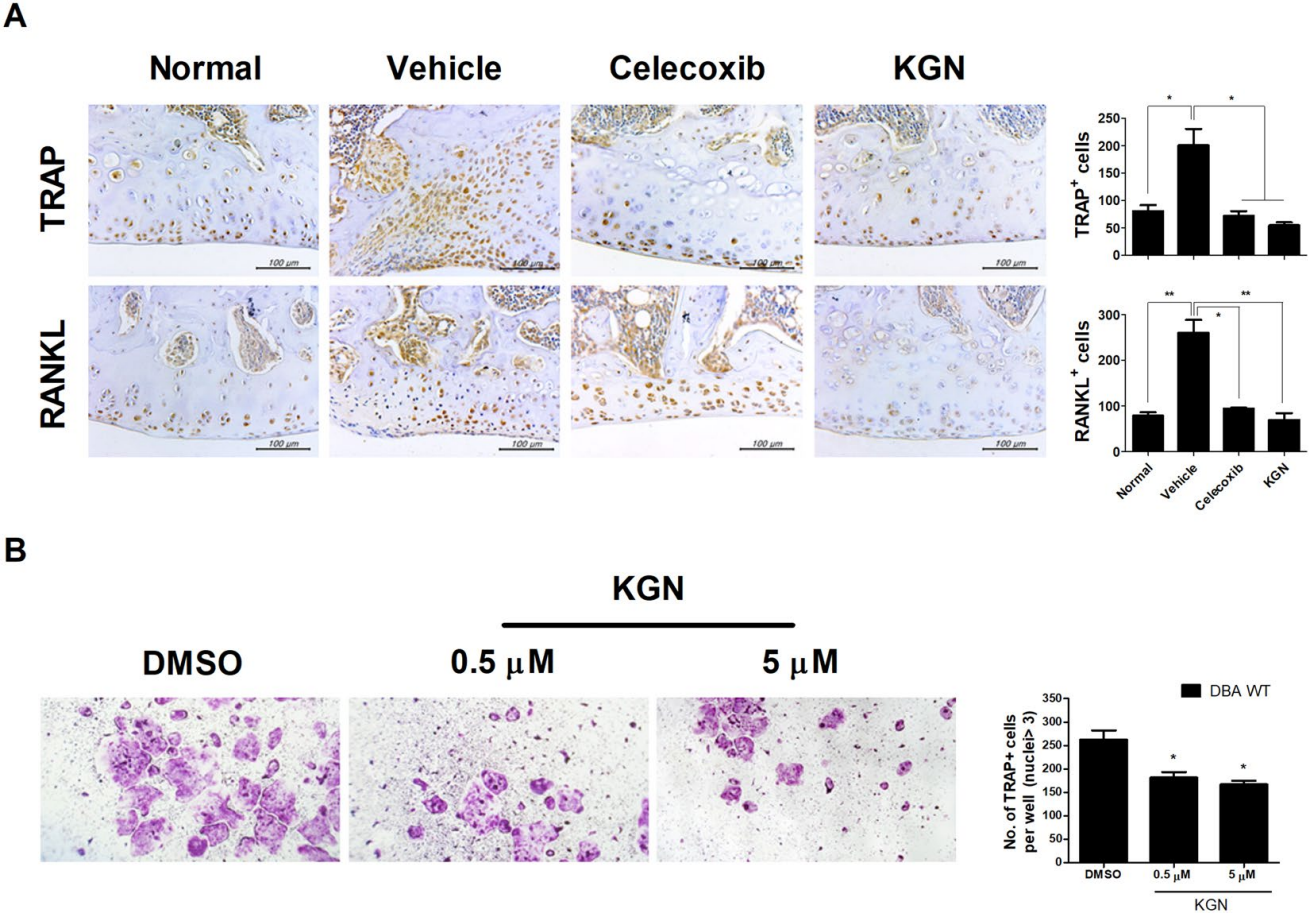
Histological evaluation of the reduction of chondroclast/osteoclast formation and activity after KGN treatment in the rat MIA model of OA. (**A**) IHC staining was analyzed to measurement the level of chondroclast factors in bone and cartilage. (**B**) Monocytes isolated from joints of DBA1/J (DBA) MIA mice and control mice were cultured with 10 ng/mL M-CSF and 0.5 or 5 μM KGN or 50 ng/mL RANKL. After maximum of 14 days of culture, TRAP-positive multinucleated cells were counted (original magnification ×10). Tissue analysis was carried out two times in three animal models. These experiments were performed in 5 individual animal experiments. (*P < 0.05, **P < 0.001, ***P < 0.005, compared to the DMSO-treated group).

The expression of TRAP and RANKL in joints and cartilage of OA rats was decreased by treatment with KGN and celecoxib (Fig. 5A). To assess the effect of KGN on RANKL-induced chondroclast differentiation, bone marrow-derived monocytes (BMMs) were cultured with KGN in the presence of M-CSF and RANKL for 14 d. KGN significantly suppressed the formation of TRAP^+^ multinuclear mature chondroclasts, in a dose-dependent manner (Fig. 5B). These results suggest that KGN has an inhibitory effect on RANKL-mediated chondroclast differentiation and activation.

### KGN improved IL-10 expression and Treg cell differentiation

**Figure 6:**
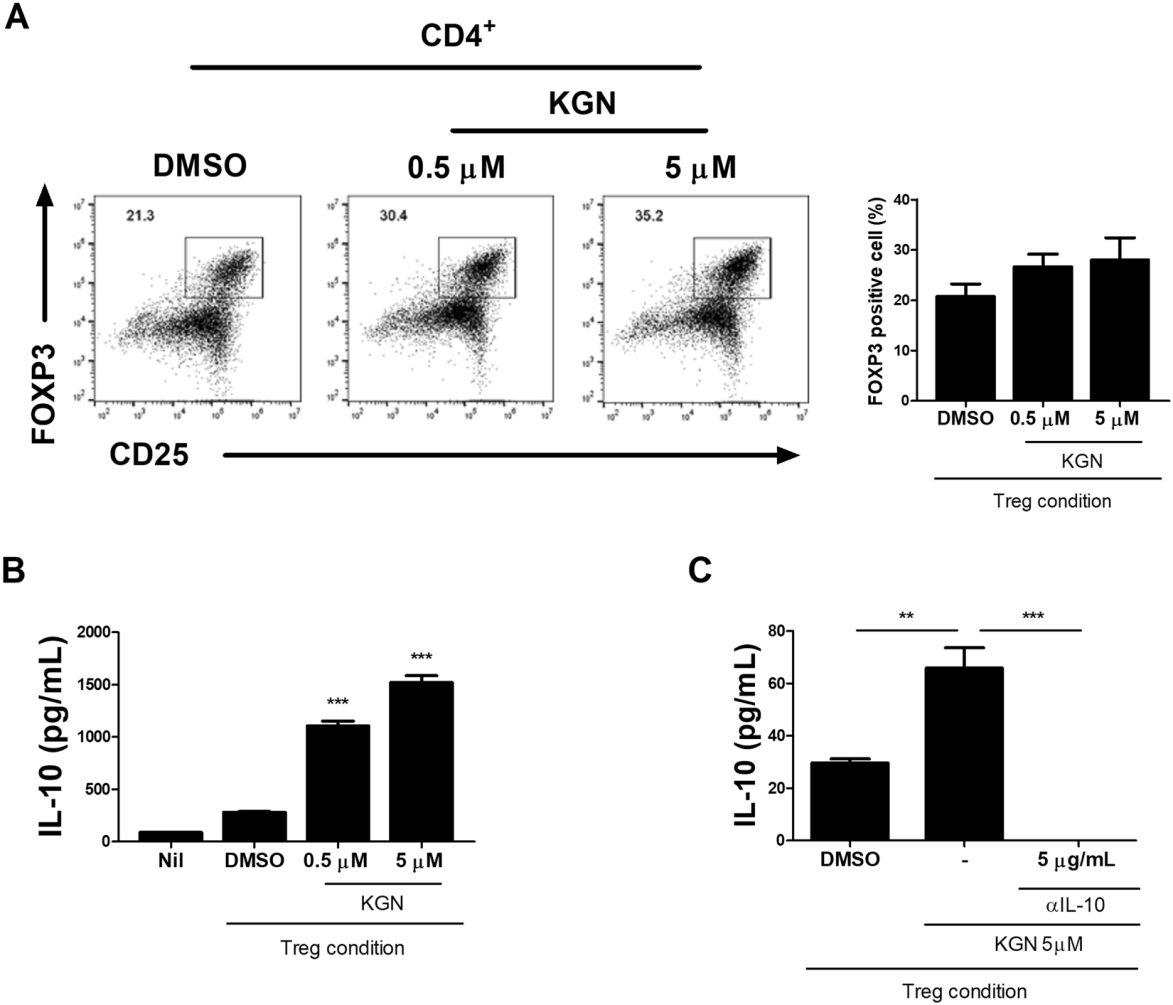
KGN enhances the expression of FOXP3 and anti-inflammatory cytokine IL-10 in T regulatory (Treg)-cells in MIA-induced OA mice. (**A**) The cells were stained with anti-mCD4-FITC-Cy5.5, anti-mCD25-APC, or anti-mFOXP3-PE. Positive Treg cells are presented in the form of a graph (CD4^+^, CD25^+^, and FOXP3^+^ cells). (**B**) Production of IL-10 by Treg-polarizing CD4^+^, CD25^+^, and FOXP3^+^ cells and secretion into the culture supernatant detected by ELISA. **P < 0.001, ***P < 0.005.

OA patients have reduced IL-10 expression and Treg cell differentiation^10^. We analyzed Treg cell differentiation and the anti-inflammatory response in splenocytes from MIA-induced DBA 1/J (DBA) mice. KGN treatment enhanced the expression of FOXP3in Treg cells (Fig. 6A) and upregulated Treg cell-generated IL-10 expression significantly (Fig. 6B). These results suggest that KGN could reduce OA severity by promoting Treg cell differentiation and IL-10 production.

### IL-10 protected against pain and KGN regulated IL-10-dependent pain suppression in the OA mice model

**Figure 7:**
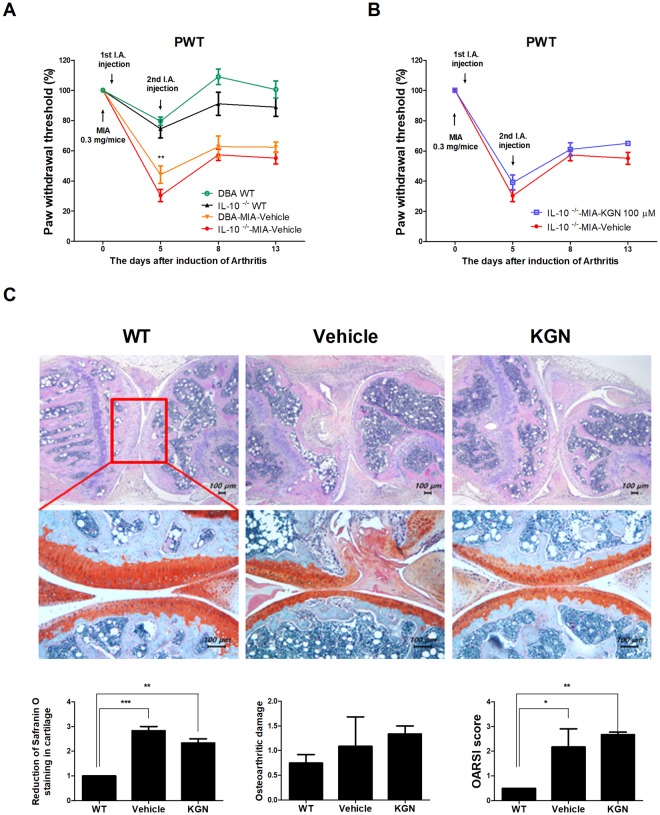
KGN deregulates pain in MIA-induced IL-10-knock-out (IL-10^−/−^) OA mice. (**A**,**B**) Conducting examination of tactile allodynia was estimated from the ugo basile dynamic plantar. (Number of the MIA or KGN treated mice = 4) PWT was determined on the injection paw. Celecoxib was applied orally every day for 13 days and mice were treated with intra-articular injection of KGN (100 μM) or vehicle 1 and 5 days after MIA injection in DBA mice. IL-10^−/−^ mice are compared with IL-10^−/−^ vehicle mice. (**C**) Safranin-O/fast green and H&E staining of the knee joints tissue was performed to analyze tissue damage. These experiments were performed in three individual animal experiments. *P < 0.05, **P < 0.001, ***P < 0.005.

Since we found that KGN upregulated anti-inflammatory cytokines such as IL-10 in chondrocytes, we examined the function of KGN in regulating degeneration of articular cartilage and suffering of joint in murine model. We additionally performed chemical hyperalgesia in osteoarthritis DBA1/J and IL-10-knock-out (IL-10^−/−^) mice. We confirmed that the mice were IL-10^−/−^ mice via ELISA (Supplementary Fig. 2). On days 1 and 5 after IA injection of MIA (0.3 mg/mouse), KGN was injected intra-articularly in the right joint of mice. MIA-induced IL-10^−/−^ mice had decreased PWL compared to the group of DBA1/J mice. MIA-induced IL-10^−/−^ mice also appeared to have more pain severity than MIA-induced DBA mice (Fig. 7A). However, KGN treatment improved pain progression in MIA-induced IL-10^−/−^ mice (Fig. 7B). DRG was destructed in MIA-treated IL-10 knockout mice while the morphology of normal status was maintained in DRG of MIA-treated DBA/1J mice^12^ (Supplementary Fig. 3). Moreover, KGN treatment decreased cartilage destruction and OARSI score^13,14^, although not significantly (Fig. 7C). These results suggest that IL-10 deficiency exacerbated pain progression and that KGN regulated MIA-induced severity in an IL-10-dependent manner.

## Discussion

Although the small molecule KGN has been studied in chondrogenesis and cartilage tissue repair, there is little proof of the beneficial function of KGN the osteoarthritis-reported medical state. In this research, we demonstrated suppression of pro-inflammatory cytokine expression and chondroclast inhibition mediated by KGN in an experimental OA rat model.

The most significant effect of KGN is anti-inflammatory activity through the upregulation of IL-10, a typical anti-inflammatory cytokine, *in vitro* and *in vivo*. It has been demonstrated that IL-10 reduces inflammatory responses and the expression of MMPs and proinflammatory cytokines such as TNF-α^15,16^. It is already well documented that IL-10 inhibits osteoclastogenesis^13,14^. Since OA pathogenesis involves the upregulation of MMPs, the inflammatory response, and osteoclasts^5,17–19^, IL-10 expression mediated by KGN could show therapeutic activity in OA. Our observations revealed that KGN treatment induces IL-10 production and has protective activity against cartilage degeneration through the downregulation of MMPs, inflammation, and chondroclasts. We also found that osteoclastogenesis is decreased by KGN *in vitro*. Thus, KGN could potentially ameliorate OA progression and prevent cartilage degeneration.

Recently, it was also reported that IL-10 expression and Treg cell differentiation were reduced in OA patients^10^. Specifically, IL-10 release of Treg cell differentiation and IL-10 production from Treg cells were reduced significantly in OA patients compared to non-OA subjects^20^. Indeed, suppression of Treg cell differentiation is involved in OA pathogenesis^21^. We found that Treg cell differentiation and IL-10 expression were increased by KGN treatment. Since downregulation of Treg cell differentiation is associated with the pathogenesis of OA, KGN may also reduce OA severity by the promotion of Treg cell differentiation and IL-10 production.

The degeneration of cartilage is associated with primary clinical symptoms in the OA joint. It is well documented that inflammation is a key factor in OA pathogenesis because it exacerbates pain and joint destruction^15^. There are also many reports that IL-10 improves pain severity and that IL-10 can be used for pain therapy^22–24^. In the present investigation, the expression of pain molecules, such as CGRP and MCP-1, was decreased by KGN treatment in dorsal root ganglion (DRG) of MIA-treated rats. KGN also regulated the reciprocal balance between proinflammatory cytokine production, including interleukin-10 and -1β, in joints in experimental osteoarthritis murine model. These results suggest that KGN could also be used to improve OA- mediated pain by downregulating the inflammatory response.

Dysregulation between anabolic and catabolic factors is associated with cartilage destruction in the pathogenesis of OA. It has been demonstrated that MMPs accelerate cartilage degeneration and are increased by OA development, whereas TIMPs improve cartilage degradation through the inhibition of MMPs^25,26^. IL-10 expression has also been found to decrease the expression of MMPs which could reduce OA development and cartilage degradation^15^. We observed that IL-1β-stimulated gene expression of catabolic mediators decreased, but the IL-10 level was enhanced by KGN in OA patients’ chondrocytes. Moreover, the expression of MMP3 and MMP13 was reduced, whereas IL-10 and TIMP3 expression was promoted by KGN treatment in MIA rat joints. These data demonstrate that KGN could exert a chondroprotective effect through enhancement of IL-10 and TIMPs in OA.

While KGN has been sown to improve experimental OA through upregulation of chondrogenesis, few have studied its role in anti-inflammatory activity and pain relief. The function of KGN demonstrated in this study identified that it likely makes an important treatment in the inhibition of osteoarthritic joint. We suggest that KGN reduces pain, catabolic activity, and inflammation through the upregulation of IL-10 in an experimental OA rat model. In conclusion, KGN is a viable therapeutic candidate for OA therapy.

## Methods

### Animals

Male rats (Wistar) aged 6 weeks were purchased from Central Lab (Seoul, Korea, Animal Inc.) and weight of 230–250 grams were applied at the initiate of the experiment. The rats were housed 3 animals in case, in a rat house with management 21–22 °C of temperature conditions, and illumination (12/12 hours, cycle of day/night). Animals fed food and water ad-libitum feeding. The Animal Research Ethics Committee of the Catholic University of Korea evaluated and approved all animal processes. (Approval Number: 2016-0002-01). All surgeries were performed under isoflurane anesthesia and all efforts were conducted to minimize suffering. Rats were monitored three times per week and euthanized in a carbon dioxide (CO_2_) chamber. No animals exhibited severe signs of illness or death due to the experimental procedures.

Thirteen-week old male DBA1/J IL-10-knockout mice (SLC, Inc., Pohang University of Science & Technology) were maintained in cohorts of five mice. They were held in polycarbonate cages in a specific pathogen-free environment and were fed standard mouse chow (Ralston Purina, Gray Summit, MO, USA) and water *ad libitum*. All experimental procedures were examined and permitted by the Animal Care Ethics Committee at the Catholic university of Korea.

### Method of OA induction in rats

Rats were randomly selected and placed in treatment groups prior to the study. For induction of OA, the right knee of the rats were injected MIA (3 mg/rat, Sigma, St. Louis, MO) into the patellar ligament of knee using a syringe fitted with a 26.5-G needle.

Experiment of OA was induced by intra articular injection of MIA (0.3 mg/10 μL) in mice, using a Hamilton syringe in the right knees.

### Schedule of KGN and celecoxib treatment

On days 1 and 5 after IA injection of MIA, KGN was introduced by IA injection into the right joint of the rats. On day 1 after the injection of MIA, celecoxib was administered daily for 14 d (Fig. 1A).

### Assessment of pain behavior

Mechanical sensitivity was used to assess pain behavior as previously described^27^. Iodoacetate injection was randomly selected to all group of animal and the Ugo Basile Dynamic Plantar (aesthesiometer, Comerio, Italy). Experiment is necessary using the von Frey hair assessment process was used to measure mechanical sensitivity. Pain behavior scores were evaluated by published standard^27^.

MIA-treated mice were tested for their response to mechanical stimulation of the masseter hind paw using a rigid von Frey filament, coupled with a force transducer (Electrovonfrey, model No: 2290, IITC Inc. Woodland Hills, CA). The force needed to elicit a withdrawal of either of the hind paws was recorded following three stimuli presentations at approximately one-minute intervals. The mean values of the three readings were used for analysis.

### Human chondrocyte separation and differentiation

The study was passed by the institutional inspection board of Uijeongbu St. Mary’s Hospital (UC14CNSI0150). All five patients who volunteered for the investigation were from Uijeongbu St. Mary’s Hospital and were fully informed on the investigation. Written agreement was acquired from all patients with OA, who performed the criteria defined by the American College of Rheumatology. To isolate chondrocytes, we obtained cartilage samples from OA patients during joint replacement surgery. The chondrocytes were isolated using published method^27^. Human chondrocyte cells were seeded in 24 well plates at a density of 5 × 10^4^. The chondrocytes were treated by KGN and 20 ng/mL human recombinant IL-1β (R&D Systems, Minneapolis, MN) for 2 days.

### Ethics statement

The experimental protocol was allowed by the Animal Research Ethics commission at the Catholic University of Korea. All experimental procedures were carried out in accordance with the approved protocols. All procedures performed followed the ethical guidelines for animal studies. Cartilage tissue collection was allowed by the institutional screening commission of Uijeongbu St. Mary’s Hospital and fulfilled in accordance with the declaration of Helsinki II. The total subjects’ information access was limited to protect volunteers’ privacy and anonymity.

### Isolation of splenocytes and culture

**S**pleens were collected for cell preparation from DBA1/J mice. The spleens were sieved and the RBCs (red blood cells) were isolated with hypotonic ammoniumchloride-potassium buffer (0.83%). Total splenocyte fraction was filtered through a cell strainer and used centrifugation (at 1,300 rpm, 4 °C for five minutes). Treg cells sere stimulated with plate-bound mAb anti-CD3 (0.5 μg/mL) (BD Biosciences, CA, USA), soluble mouse antibodies anti-CD28 (1 μg/mL, BD Biosciences), 2 μg/mL of anti-IFN-γ, interleukin-4 antibodies and 5 ng/mL recombinant TGF-β (transforming growth factor β, all from R&D Systems) for 3 d.

### Gene expression analysis using real-time PCR

Whole ribonucleic acid (RNA) was separated from chondrocytes utilizing the TRI reagent protocol (Invitrogen). cDNA (complimentary DNA) was arranged by reverse transcription of the single-stranded ribonucleic acid per the producer’s instructions involved in the High Capacity complimentary DNA Reverse Transcription Kit (Applied Biosystems). Messenger RNA (mRNA) were analyzed by real-time PCR with Light-Cycler FastStart deoxyriboNucleic acid (DNA) Master SYBR Green I kit (Takara) in accordance with the producer’s directions. The following primers were utilized in sequences: Control human gene β-actin, 5′-GGA CTT CGA GCA AGA GAT GG-3′ (sense) and 5′-TGT GTT GGC GTA CAG GTC TTT G-3′ (antisense); Human tissue inhibitor of metalloproteinase 3 (TIMP3), 5′-CTG ACA GGT CGC GTC TAT GA-3′ (sense) and 5′-GGC GTA GTG TTT CTG GT-3′ (antisense); Human TIMP1, 5′-AAT TCC GAC CTC GTC ATC AG-3′ (sense) and 5′-TGC AGT TTT CCA GCA ATG AG-3′ (antisense); Human matrix metalloproteinase 3 (MMP3), 5′-CTC ACA GAC CTG ACT CGG TT-3′ (sense) and 5′-CAC GCC TGA AGG AAG AGA TG-3′ (antisense); and human interleukin-10 (IL-10), 5′-CCA AGC CTT GTC TGA GAT GA-3′ (sense) and 5′-TGA GGG TCT TCA GGT TCT CC-3′ (antisense). All expression values were normalized to that of β-actin messenger RNA (mRNA). PCR amplification and analysis were performed using Light-Cycler Real time polymerase chain reaction (PCR) system (Roche Diagnostics).

### FACS (Flow cytometry) analysis

Mononuclear cells were immunostained with various combinations of fluorescent antibodies against CD4, CD25, and forkhead box P3 (FOXP3) (eBiosciences, San Diego, CA, USA). Intracellular staining was performed using a Foxp3/Transcription Factor Staining Buffer kit (eBiosciences) per the manufacturer’s protocol. FACs analysis was conducted a CytoFLEX flow cytometer (Beckman Coulter, USA).

### Sandwich ELISA

The concentrations of cytokines were measured by sandwich enzyme-linked immunosorbent assay. Abs directed against human IL-10 and biotinylated anti-human IL-10 (R&D Systems) were used as the capture and detection antibodies, respectively. The concentration of cytokines present in the test samples was determined from standard curves established with serial dilutions of recombinant IL-10 (R&D Systems). The absorbance at 450 nm was measured using an ELISA microplate reader (Molecular Devices, Sunnyvale, CA, USA).

### Histopathological analysis

Rat joint tissues were obtained two weeks post-immunization. Joint tissues were sectioned at 7-μm thickness, dewaxed using xylene, dehydrated through an alcohol gradient, and stained with Safranin O and H&E (hematoxylin and eosin).

### Immunohistochemistry

The slides were treated with H_2_O_2_ in methanol to eliminate endogenous peroxidase activity and fixed with NGS (normal goat serum) for thirty minutes. The slides were incubated with the first primary monoclonal antibodies overnight at 4 °C; the antibodies were for TNF-α, IL-6, IL-10, IL-1β, calcitonin gene-related peptide (CGRP), monocyte chemoattractant protein (MCP)-1, receptor activator of nuclear factor kappa-B ligand (RANKL), MMP3, MMP13, TIMP3 (Abcam, Cambridge, UK), transient receptor potential cation channel subfamily V member (TRPV)-1 (R&D Systems), C-C chemokine receptor type (CCR)-2 (Novus Biologicals, USA), and TRAP (Santa Cruz Biotechnology). And sections were bound to each secondary Abs (antibodies). First primary Abs were detected with a biotinylated secondary linking Ab, followed by incubation with streptavidin-peroxidase complex for 1 h. The product was developed using 3, 3′-diaminobenzidine chromogen (Dako, Carpinteria, CA). Positive cells were counted and the results are expressed as mean ± standard deviation (SD).

### *In vivo* micro-CT imaging and analysis

Micro-CT imaging and analysis were performed using a bench-top cone-beam type *in vivo* animal scanner (SKYSCAN1172 micro-CT, Bruker micro CT, Belgium), a method that has been described previously in detail [19, 20]. Briefly, rats were sacrificed with a 9:1 mixture of ketamine and Rompun (5 mL/kg). The hind limbs of the dead rats were dissected and immediately fixed in 10% neutral buffered formalin 2 weeks after the injection. Samples were imaged with settings of 70 kVp, 141 μA, and Aluminum 0.5-mm thick filter. The pixel size was 8.0 μm and the rotation step was 0.4°. The cross-sectional images were reconstructed using a filtered back-projection algorithm (NRecon software, Bruker micro CT, Belgium). For each scan, a stack of 286 cross-sections was reconstructed at 2,000 × 1,335 pixels. Bone mineral density was calculated at the lateral femoral condyle area of each sample. A semi-quantitative method using the degree of osteophytes and joint destruction was introduced to grade the degree of OA changes.

### Statistical analysis

All data were expressed as the mean ± standard deviation (SD). Statistical analyses was performed using GraphPad Prism (ver.5.01) software and conducted using ANOVA (one-way analysis of variance) with Bonferroni’s post-test for multiple comparisons. A *P*- value less than 0.05 was considered statistically significant.

## Electronic supplementary material

Dataset 1

## References

[1] Gupta S, Hawker GA, Laporte A, Croxford R, Coyte PC (2005). The economic burden of disabling hip and knee osteoarthritis (OA) from the perspective of individuals living with this condition. Rheumatology.

[2] Bitton R (2009). The economic burden of osteoarthritis. The American journal of managed care.

[3] Kapoor M, Martel-Pelletier J, Lajeunesse D, Pelletier JP, Fahmi H (2011). Role of proinflammatory cytokines in the pathophysiology of osteoarthritis. Nature reviews. Rheumatology.

[4] Burrage PS, Mix KS, Brinckerhoff CE (2006). Matrix metalloproteinases: role in arthritis. Frontiers in bioscience: a journal and virtual library.

[5] Yuan XL (2014). Bone-cartilage interface crosstalk in osteoarthritis: potential pathways and future therapeutic strategies. Osteoarthritis and cartilage.

[6] Alam MR, Ji JR, Kim MS, Kim NS (2011). Biomarkers for identifying the early phases of osteoarthritis secondary to medial patellar luxation in dogs. Journal of veterinary science.

[7] Jhun J (2015). The chicken combs extract alleviates pain and cartilage degradation in rat model osteoarthritis. Tissue Engineering and Regenerative Medicine.

[8] Kaneko S (2000). Interleukin-6 and interleukin-8 levels in serum and synovial fluid of patients with osteoarthritis. Cytokines Cell Mol Ther.

[9] Kammermann JR, Kincaid SA, Rumph PF, Baird DK, Visco DM (1996). Tumor necrosis factor-alpha (TNF-alpha) in canine osteoarthritis: Immunolocalization of TNF-alpha, stromelysin and TNF receptors in canine osteoarthritic cartilage. Osteoarthritis Cartilage.

[10] Penatti A (2017). Differences in serum and synovial CD4+ T cells and cytokine profiles to stratify patients with inflammatory osteoarthritis and rheumatoid arthritis. Arthritis research & therapy.

[11] Johnson K (2012). A stem cell-based approach to cartilage repair. Science.

[12] Marshall J (2010). Substrate reduction augments the efficacy of enzyme therapy in a mouse model of Fabry disease. Plos One.

[13] Xu LX (1995). Interleukin-10 selectively inhibits osteoclastogenesis by inhibiting differentiation of osteoclast progenitors into preosteoclast-like cells in rat bone marrow culture system. Journal of cellular physiology.

[14] Evans KE, Fox SW (2007). Interleukin-10 inhibits osteoclastogenesis by reducing NFATc1 expression and preventing its translocation to the nucleus. BMC cell biology.

[15] Wojdasiewicz P, Poniatowski LA, Szukiewicz D (2014). The role of inflammatory and anti-inflammatory cytokines in the pathogenesis of osteoarthritis. Mediators Inflamm.

[16] Shin DI (1999). Interleukin 10 inhibits TNF-alpha production in human monocytes independently of interleukin 12 and interleukin 1 beta. Immunological investigations.

[17] Karsdal MA (2014). The coupling of bone and cartilage turnover in osteoarthritis: opportunities for bone antiresorptives and anabolics as potential treatments?. Annals of the rheumatic diseases.

[18] Berenbaum F (2013). Osteoarthritis as an inflammatory disease (osteoarthritis is not osteoarthrosis!). Osteoarthritis and cartilage.

[19] Blasioli DJ, Kaplan DL (2014). The roles of catabolic factors in the development of osteoarthritis. Tissue Eng Part B Rev.

[20] Li S (2016). Downregulation of IL-10 secretion by Treg cells in osteoarthritis is associated with a reduction in Tim-3 expression. Biomedicine & pharmacotherapy = Biomedecine & pharmacotherapie.

[21] Li YS, Luo W, Zhu SA, Lei GHT (2017). Cells in Osteoarthritis: Alterations and Beyond. Frontiers in immunology.

[22] Plunkett JA, Yu CG, Easton JM, Bethea JR, Yezierski RP (2001). Effects of interleukin-10 (IL-10) on pain behavior and gene expression following excitotoxic spinal cord injury in the rat. Experimental neurology.

[23] Milligan, E. D., Penzkover, K. R., Soderquist, R. G. & Mahoney, M. J. Spinal interleukin-10 therapy to treat peripheral neuropathic pain. *Neuromodulation: journal of the International Neuromodulation Society* 15, 520–526; discussion 526, 10.1111/j.1525-1403.2012.00462.x (2012). 10.1111/j.1525-1403.2012.00462.x PMC3443506 22672183

[24] Ogawa N (2014). Gene therapy for neuropathic pain by silencing of TNF-alpha expression with lentiviral vectors targeting the dorsal root ganglion in mice. Plos one.

[25] Mueller MB, Tuan RS (2011). Anabolic/Catabolic balance in pathogenesis of osteoarthritis: identifying molecular targets. PM R.

[26] Kashiwagi M, Tortorella M, Nagase H, Brew K (2001). TIMP-3 is a potent inhibitor of aggrecanase 1 (ADAM-TS4) and aggrecanase 2 (ADAM-TS5). J Biol Chem.

[27] Jeong JH (2015). Eupatilin Exerts Antinociceptive and Chondroprotective Properties in a Rat Model of Osteoarthritis by Downregulating Oxidative Damage and Catabolic Activity in Chondrocytes. Plos One.

