# Supplementary material for: Kartogenin inhibits pain behavior, chondrocyte inflammation, and attenuates osteoarthritis progression in mice through induction of IL-10
Source: Sci Rep. 2018 Sep 14;8:13832. doi: 10.1038/s41598-018-32206-7 (PMC6138726; doi:10.1038/s41598-018-32206-7)
Supplement: Supplementary file 1 — Dataset 1 [file 41598_2018_32206_MOESM1_ESM.pdf]

Supplemental Information

Kartogenin inhibits pain behavior, chondrocyte inflammation, and attenuates osteoarthritis progression in mice through induction of IL-10

Ji Ye Kwon[1], Seung Hoon Lee[1], Hyun-Sik Na[1], KyungAh Jung[2], JeongWon Choi[1], Keun Hyung Cho[1], Chang-Yong Lee[3], Seok Jung Kim[4], Sung-Hwan Park[1], Dong-Yun Shin[3*], and Mi-La Cho[1,2*]

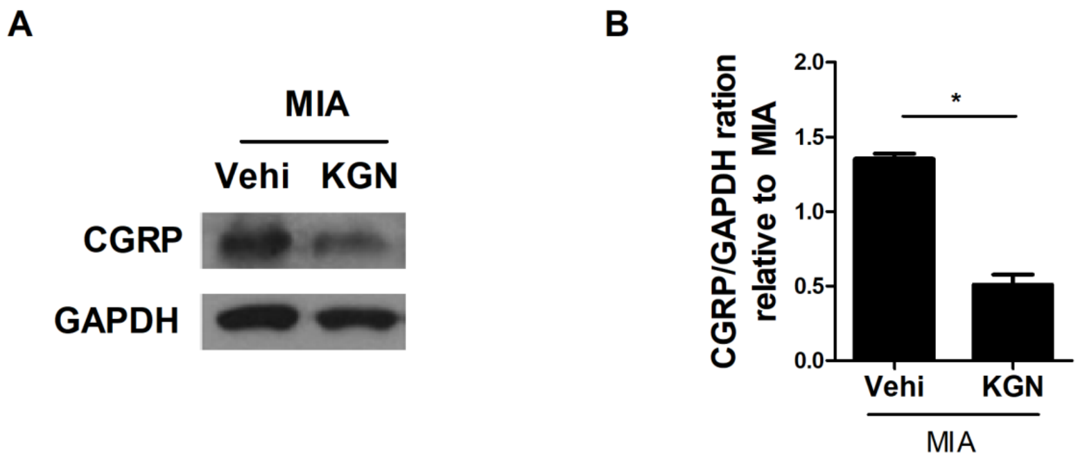

Supplementary Figure 1: The expression of CGRP decreased in dorsal root ganglion of KGN-treated rats compared to MIA-treated rats. CGRP expression in dorsal root ganglion of rats was evaluated by Western blot. This result acquired of same blots. The level of CGRP is expressed as a relative ratio and GAPDH was used to normalize the data. (*$P < 0.05$, **$P < 0.001$, ***$P < 0.005$)

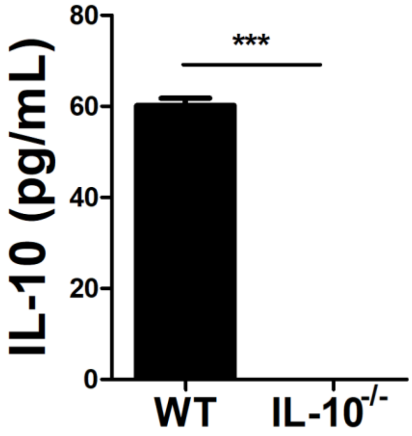

Supplementary Figure 2: IL-10 production in mice splenocytes from WT (DBA/1J) and IL-10-knock out to confirm IL-10 KO efficiency. The expression of IL-10 was analyzed by ELISA using splenocytes from WT and IL-10 KO cultured for 24 hours under LPS 100 ng/mL treatment. (*$P < 0.05$, **$P < 0.001$, ***$P < 0.005$)

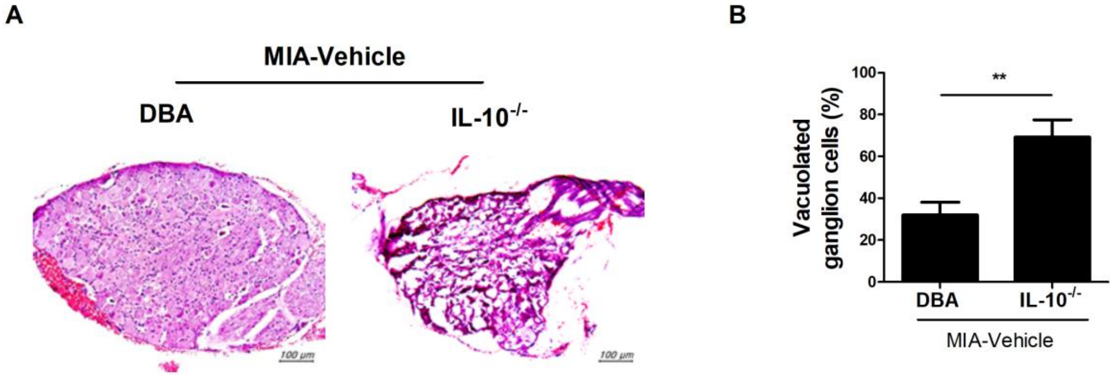

Supplementary Figure 3: Vacuolated ganglion cells were increased in IL-10 knockout mice.

Quantification of vacuolated ganglion cells in percent is presented in the graph. Scale bar, 200 μm. (*$P < 0.05$, **$P < 0.001$, ***$P < 0.005$)
